# Comprehensive utilization of edible mushroom *Auricularia auricula* waste residue—Extraction, physicochemical properties of melanin and its antioxidant activity

**DOI:** 10.1002/fsn3.1239

**Published:** 2019-10-21

**Authors:** Xin Liu, Ruolin Hou, Danting Wang, Mengxian Mai, Xiaoping Wu, Mingfeng Zheng, Junsheng Fu

**Affiliations:** ^1^ College of Food Sciences Fujian Agriculture and Forestry University Fuzhou China; ^2^ College of Life Sciences Fujian Agriculture and Forestry University Fuzhou China; ^3^ Mycological Research Center College of Life Sciences Fujian Agriculture and Forestry University Fuzhou China

**Keywords:** antioxidant activity, *Auricularia auricula*, melanin, physicochemical properties, waste residue

## Abstract

In order to promote the comprehensive utilization of the *Auricularia auricula* waste residue, the extraction process and the physicochemical properties of melanin from *A. auricula* waste residue were studied. Furthermore, the chemical antioxidant activity of waste residue melanin and its protective effect on cell oxidative injury induced by H_2_O_2_ were investigated. The results indicated that the ultrasonic‐assisted extraction process could be used to extract the melanin from *A. auricula* waste residue. Melanin had a good solubility in alkali solution and exhibited a certain stability to thermal. There was no significant difference between *A. auricula* melanin control group and waste residue melanin on ABTS, DPPH, and hydroxyl radical scavenging activity. Waste residue melanin significantly inhibited the cell death caused by H_2_O_2_, and the cell viability was restored to 98.09 ± 5.97% when the melanin concentration was 1.6 mg/ml. Cell morphology observation confirmed that the melanin ameliorated the morphological changes of cells induced by oxidative stress.

## 1 INTRODUCTION

Pigment is an important part of food. At present, the safety of synthetic pigments has been paid more and more attention, and the application of natural pigments has aroused people's interest. In addition to create the ideal color, natural pigments can also be used as functional ingredients (Li, Zhang, & Liu, 2013). Melanin is a kind of irregular pigment that composed of phenolic or indolic monomers, existing in animals, plants, and microorganisms (Huang et al., 2018). Melanin has many biological activities, such as eliminate free radical (Kim et al., 2017), antitumor (Shi et al., 2018), and anti‐radiation (Ye et al., 2014).

*Auricularia auricula* is a popular edible mushroom in East Asia, especially in China, Japan, and Korea (Nguyen, Chen, et al., 2012; Sękara, Kalisz, Grabowska, Kalisz, Grabowska, & Siwulski, 2015). Modern pharmacological studies have shown that *A. auricula* have many biological activities such as antioxidant (Xu et al., 2018), improve immunity (Nguyen, Wang, et al., 2012), and antitumor (Ma et al., 2018). Growing concerns over the huge amount of substrate wastes of *A. auricula* to the squander of resources and the contamination of environmental comes with the boom of *A. auricula* industry. At present, most researches have focused on the extraction and bioactivity of the polysaccharides from *A. auricular* (Bao et al., 2017; Qiu, Zhang, Wang, Zhang, Wang, Liu, & Regenstein, 2016; Xu et al., 2018). However, large amount of *A. auricula* waste residue will be produced during the extraction process of polysaccharides, and there is no good way to deal with these waste residues. Due to the special properties of melanin that are insoluble in water and organic solvents (Madhusudhan, Mazhari, Dastager, Mazhari, Dastager, & Agsar, 2014; Sun, Zhang, Chen, Zhang, Chen, Zhang, & Zhu, 2016), melanin still remains in the waste residues of *A. auricula* fruit body after extracting polysaccharides and other active substances. It has been reported that *A. auricula* melanin has good hepatoprotective (Hou et al., 2019) and antimicrobial activities (Bin, Wei, Xiaohong, Wei, Xiaohong, Mei, & Mingsheng, 2012). Thus, extracting the melanin from the waste residues of *A. auricula* can promote the comprehensive utilization and expand the application value of *A. auricula*.

In order to make the best use of the various active ingredients of *A. auricula*, the waste residue of *A. auricula* fruiting body after extraction of polysaccharide was selected as raw material to study the extraction process of melanin. Furthermore, the physical and chemical properties and antioxidant activity of the melanin were explored. This work is of certain significance to the comprehensive utilization of *A. auricula* and also provides a reference for the utilization of the other edible fungus waste residue resources.

## 2 MATERIALS AND METHODS

### 2.1 Materials and chemicals

*Auricularia auricula* waste residue powder was obtained by drying the powder of fruiting body after the polysaccharide was extracted. Human L02 hepatocyte cells were purchased from Fuheng Biological Technology Co.. ABTS radical and DPPH radical were purchased from Sigma‐Aldrich. Chemical reagents such as sodium hydroxide, hydrochloric acid, ethyl acetate, chloroform, ethanol, salicylic acid, ferrous sulfate, and hydrogen peroxide were purchased from Sinopharm Co..

### 2.2 Extraction procedure of melanin from *Auricularia auricula* waste residue

*Auricularia auricula* waste residue was added into a NaOH solution and placed in an ultrasonic bath with temperature and power control (PS‐G60, JieKang Ultrasonic Equipment) to investigate the effects of various factors on the yield of melanin. After the extraction process was completed, the supernatant was obtained by centrifugation, and the melanin was purified according to the method previous reports (Sun, Zhang, Sun, et al., 2016; Zou, Hu, Ma, Hu, Ma, & Tian, 2015). The pH of the supernatant was adjusted to 1.5 with 6 mol/L HCl and kept in a 80°C water bath for 10 hr. The precipitate was collected by centrifugation, and the precipitate was washed with deionized water until neutral to obtain crude melanin. The crude melanin was redissolved in a 1.5 mol/L NaOH solution, and the insoluble impurities were removed by centrifugation. The pH of the supernatant was adjusted to 1.5 with 6 mol/L HCl and stored at 4°C for 5 hr. The precipitate was collected by centrifugation and washed to neutral with deionized water. Pure melanin was obtained by rinsing successively with chloroform, dichloromethane, ethyl acetate, absolute ethanol and deionized water, and then freeze‐drying. The maximum absorption wavelength of the purified waste residue melanin was determined by an ultraviolet‐visible spectrophotometer to be 215 nm. By measuring the absorbance of the melanin at 215 nm, a standard curve corresponding to the relationship between the mass concentration and the absorbance was plotted. The concentration of the melanin was calculated from the standard curve, and the melanin yield under each extraction condition was calculated according to Equation (1), the weight of the melanin extract is represented by *w_0_*, and the weight of *A. auricula* waste powder is represented by *M*.

(1) $$\text{The}\;\text{yield}\;\text{of}\;\text{melanin}\;\left(\%\right)\;=\;\frac{w_{0}}{M}\;\times \;100$$

### 2.3 Experimental design

The effects of solid–liquid ratio (1:10 ~ 1:60), ultrasonic power (200 ~ 500 W), ultrasonic time (10 ~ 80 min), ultrasonic temperature (20 ~ 80°C), and NaOH concentration (0.125 ~ 0.750 mol/L) on the yield of melanin were investigated by single‐factor experiments. Factors other than variables were kept unchanged in the experiment. The RSM experiment was carried out based on the factors that had greater influence on the yield of melanin in the single‐factor experiment.

According to the principle of Box–Behnken design (BBD), the experiment was designed by Design‐Expert 8.0.6 software to optimize the process parameters of ultrasonic‐assisted extraction of waste residue melanin. Independent variables and their encoding levels are shown in Table 1. In order to evaluate the effect of independent variables on the yield of melanin, the independent variables A (Solid–liquid ratio), B (Ultrasonic power), and C (NaOH concentration) were designed and 17 experiments were carried out. The model of a second‐order polynomial was expressed to predict the optimal point according to Equation (2), where *Y* represents the response variables, *β_0_* is the model constant, *β_i_*, *β_ii_*, and *β_ij_* are the coefficient of the linear effect, coefficient of the quadratic effect, and cross‐product coefficients, respectively, *X_i_* and *X_j_* are the independent variables affecting the response.

(2) $$Y=\beta_{0}+\sum_{i=1}^{3}\beta_{i}X_{i}+\sum_{i=1}^{3}\beta_{\mathrm{ii}}X_{i}^{2}+\sum_{i<j=1}^{3}\beta_{\mathrm{ij}}X_{i}X_{j}$$

**Table 1:** Factors and levels for Box–Behnken design

Factor	Code	Level		
		−1	0	1
Solid–liquid ratio/(g/ml)	A	1:30	1:40	1:50
Ultrasonic power/(W)	B	400	450	500
NaOH concentration/(mol/L)	C	0.500	0.625	0.750

### 2.4 Characterization studies of melanin from *Auricularia auricula* waste residue

FT‐IR analysis: The *A. auricula* waste residue melanin or *A. auricula* melanin was mixed and homogenized with KBr, respectively. The mixture of melanin and KBr was compressed and analyzed with FT‐IR (Bruker VERTEX 80) at wave numbers 4,000–400/cm.

Solubility analysis: One milligram of waste residue melanin was added into 2 ml of different organic solvents, inorganic solutions, and salt solutions. The melanin was fully mixed with the solvent and stood still for 3 hr. The solubility of melanin was determined at 215 nm.

Thermal stability analysis: *A. auricula* waste residue melanin was dissolved in 0.1 mol/L NaOH solution and incubated at 25, 50, 75, and 100°C, respectively. The samples were collected hourly, and the absorbance was measured at 215 nm, with 0.1 mol/L NaOH solution as the reference.

Light stability analysis: *A. auricula* waste residue melanin was dissolved in 0.1 mol/L NaOH solution, stored in dark, natural light and 20,000 lux strong light, respectively. Samples were collected every 12 hr to determine the absorbance at 215 nm, with 0.1 mol/L NaOH solution as the reference.

Stability in oxidants and reductants: *A. auricula* waste residue melanin solution was added to different concentrations of reducing agent (30% Na_2_SO_3_) or an oxidizing agent (30% H_2_O_2_) to make the final concentration of oxidants or reducers of 0%, 3%, 6%, 9%, 12%, and 15%. After fully mixed and left to stand for 30 min, the absorbance was measured at 215 nm, with 0.1 mol/L NaOH solution as reference. Additionally, KMnO_4_ solution with concentration of 1 mg/ml was fully mixed with waste residue melanin and allowed to stand at room temperature for 30 min; the discoloration of KMnO_4_ solution was observed.

### 2.5 Determination of chemical antioxidant activities of waste residue melanin

The water‐soluble melanin was prepared based on previously reported methods (Yang et al., 2015) and used for further studies. Waste residue melanin was dissolved in a 0.1 M NaOH solution and then adjusted pH to 7 with 0.1 m HCL solution under strong sonication. Then, water‐soluble melanin was obtained by dialysis and freeze‐drying.

The scavenging activity of *A. auricula* waste residue melanin to DPPH radical was measured on the basis of previously described methods (Wang et al., 2017). The melanin solution and 0.2 mM DPPH absolute ethanol solution were thoroughly mixed. The solution was placed in darkness for 30 min and measure the absorbance at 517 nm (*A_1_*). The same volume of absolute alcohol was substituted for the DPPH solution, and the absorbance (*A_2_*) was determined. The same volume of distilled water was substituted for the melanin solution, and the absorbance (*A_0_*) was determined. The scavenging capacity of DPPH radical was calculated according to Equation (3).

(3) $$\text{Scavenging}\;\text{activity}\;\left(\%\right)\;=\;[1\;-\;\left(A_{1}\;-\;A_{2}\right)\;/\;A_{0}]\;\times \;100$$

The ABTS radical cation (ABTS^+^) test was conducted based on previously described methods (Wang et al., 2012). A 7 mmol/L ABTS solution and 2.45 mmol/L potassium persulfate solution were mixed, and the mixture was left in the dark for 16 hr. Then, the ABTS^+^ solution was diluted with ethanol to an absorbance at 734 nm of 0.70 ± 0.02. Different concentrations of melanin solution were mixed with ABTS^+^ solution and incubated in darkness for 60 min, and the absorbance was measured at 734 nm (*A_1_*). Instead of the ABTS^+^ working solution with an equal volume of distilled water, the absorbance was measured (*A_2_*). Instead of the *A. auricula* melanin solution with an equal volume of distilled water, the absorbance was measured (*A_0_*). The ability of ABTS radical scavenging rate was calculated according to Equation (3).

The hydroxyl radical scavenging rate was determined according to the procedure previously described (Ye et al., 2015). One milliliter of 9 mmol/L salicylic acid–ethanol solution, 9 mmol/L FeSO_4_ solution, different concentrations of melanin solution, and 8.8 mmol/L H_2_O_2_ solution were thoroughly mixed and then supplemented with distilled water to 15 ml. The sample was stored at 37°C for 15 min, and the absorbance (*A_1_*) was measured at 510 nm. An equal volume of distilled water was substituted for the H_2_O_2_ solution, and the absorbance (*A_2_*) was measured. An equal volume of distilled water was substituted for the melanin solution, and the absorbance was measured (*A_0_*). The scavenging capacity of the hydroxyl radical was calculated according to Equation (3).

### 2.6 Protective effects of waste residue melanin on cell oxidative damage induced by H_2_O_2_

The cell experiment was performed according to the method previously described (Yan et al., 2018). Human L02 hepatocytes were cultured with RPMI‐1640 medium containing 10% FBS in a 37°C, 5% CO_2_ incubator. The cells in logarithmic growth stage were inoculated into a 96‐well plate at a density of 1 × 10^4^ cells/well and cultured for 24 hr. The cells were pretreated with the melanin for 1 hr and then co‐incubated with H_2_O_2_ for 24 hr. The medium containing 10% FBS was changed to medium containing 0.5% FBS at 12 hr before melanin treatment to reduce the effect of serum.

MTT assay was used to detect cell viability. After the cells were well treated, the previous culture medium was removed, and 90 µl of fresh culture medium and 10 µl of 5 mg/ml of MTT were added. After the cells were cultured at 37°C for 4 hr, the medium was removed and 150 µl of dimethyl sulfoxide (DMSO) was added to each well. The absorbance was measured at 490 nm by microplate reader. The morphology of L02 cells was observed by inverted microscope to study the effect of waste residue melanin on the morphology of cells.

### 2.7 Statistical analysis

Design‐Expert software 8.0.6 (Stat‐Ease Inc.) and SPSS 13.0 (SPSS Inc.) were used to analyze the experimental data. Experiments were repeated at least in triplicate. The result values are presented as the mean ± standard deviation, *p* < .05 indicates a statistically significant difference, and *p* < .01 indicates a highly statistically significant difference.

## 3 RESULTS AND DISCUSSION

### 3.1 Single‐factor experiment analysis

**Figure 1:**
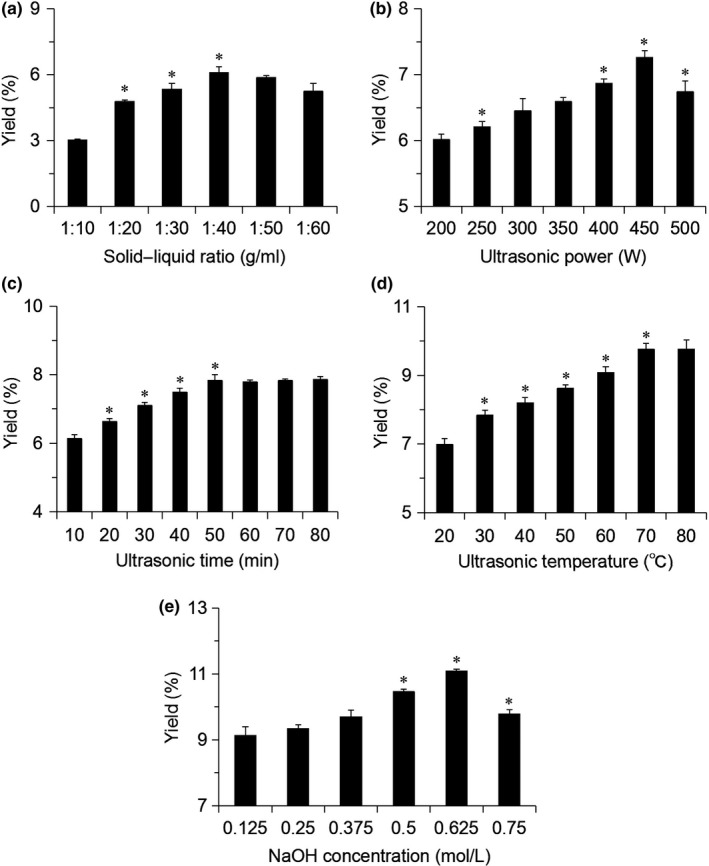
The effect of each variable on the yield of *Auricularia auricula* waste residue melanin. (a) Solid–liquid ratio; (b) ultrasonic power; (c) ultrasonic time; (d) ultrasonic temperature; and (e) NaOH concentration. Data are presented as mean ± *SD*, *p < .05 compared to the previous condition

As shown in Figure 1a, the yield of melanin gradually increased when the solid–liquid ratio was from 1:10 to 1:40. Hence, 1:40 is chosen as the center of further experiments. As shown in Figure 1b, the yield of melanin increased when the ultrasonic power increased from 200 to 450 W. When the power reached 500 W, the yield began to decrease. So, 400 ~ 500 W was selected for the further experiments. As shown in Figure 1c, the yield of melanin increased gradually when the ultrasonic time was from 10 to 50 min. After this, the yield of melanin was no longer increased. Therefore, 50 min was selected in the later RSM experiment. As shown in Figure 1d, the extraction rate of melanin increased with the increase in temperature before 70°C. However, when the temperature was higher than 70°C, the extraction rate remained unchanged. So, 70°C is a suitable temperature. As shown in Figure 1e, when the concentration of NaOH was 0.625 mol/L, the yield reached the maximum. After this, the yield began to decline. Therefore, 0.50 ~ 0.75 mol/L NaOH was chosen for the next experiment. According to the single‐factor experiment, it can be concluded that the extraction rate of melanin remained unchanged when the ultrasonic time and temperature increased to a certain extent. Therefore, in the subsequent experiment, the ultrasonic time and the ultrasonic temperature were kept constant, and the optimum solid–liquid ratio, ultrasonic power, and NaOH concentration for extracting melanin from *A. auricula* waste residue were investigated.

### 3.2 RSM model for waste residue melanin extraction process

According to the results of single‐factor experiment, the ultrasonic time of 50 min and the ultrasonic temperature of 70°C remain unchanged in the RSM experiment. A total of 17 experiments were designed and carried out by Box–Behnken design, and the experiment results are listed in Table 2. The relationship between the melanin yield (*Y*) and three variables in coding factors was described by fitting second‐order polynomial equation with linear multivariate regression method were as follows:

(4) $$\begin{array}{c} Y=11.55-0.83A+0.32B+0.25C+0.0025AB-\\ \;0.2AC-0.048BC-1.16A^{2}-0.38B^{2}-0.64C^{2} \end{array}$$

**Table 2:** Design and results of RSM

Runs	A	B	C	Melanin yield/%
1	−1	1	0	11.15
2	0	1	1	11.00
3	0	−1	1	10.57
4	1	0	−1	8.82
5	1	1	0	9.61
6	0	0	0	11.48
7	1	−1	0	8.85
8	0	0	0	11.51
9	0	0	0	11.80
10	1	0	1	8.90
11	−1	0	1	11.06
12	0	1	−1	10.57
13	0	0	0	11.47
14	0	−1	−1	9.95
15	0	0	0	11.48
16	−1	0	−1	10.20
17	−1	−1	0	10.40

The effectiveness of the fitted model was evaluated by analysis of variance (ANOVA). The results are shown in Table 3. The model *F*‐value of 91.59 and the associated lower *p*‐value (*p* < .0001) mean that the generated model is meaningful. The determination of the coefficient value (*R* ^2^) was 0.9916, and the adjusted *R* ^2^ value (Adj. *R* ^2^) was 0.9808 indicated a good correlation between the responses and independent variables. In addition, the low *F*‐value (0.35) and associated high *p*‐value (.5209) of “lack of fit” indicated the model is suitable for accurate prediction of variation (Quanhong & Caili, 2005).

**Table 3:** ANOVA of the fitted polynomial quadratic model

Source	*df*	Sum of squares	Mean squares	F	*p*
Model	9	15.73	1.75	91.59	<.0001
A	1	5.49	5.49	287.99	<.0001
B	1	0.82	0.82	42.94	.0003
C	1	0.5	0.5	25.95	.0014
A^2^	1	5.68	5.68	297.72	<.0001
B^2^	1	0.62	0.62	32.54	.0007
C^2^	1	1.73	1.73	90.82	<.0001
AB	1	2.50E−05	2.50E−05	1.31E−03	.9721
AC	1	0.15	0.15	7.97	.0256
BC	1	9.03E−03	9.03E−03	0.47	.5137
Lack of fit	3	0.053	0.018	0.35	.5209
Residual error	7	0.13	0.019	*R* ^2^ = 0.9916	
Pure error	4	0.08	0.02	Adj *R* ^2^ = 0.9808	
Total	16	15.86			

**Figure 2:**
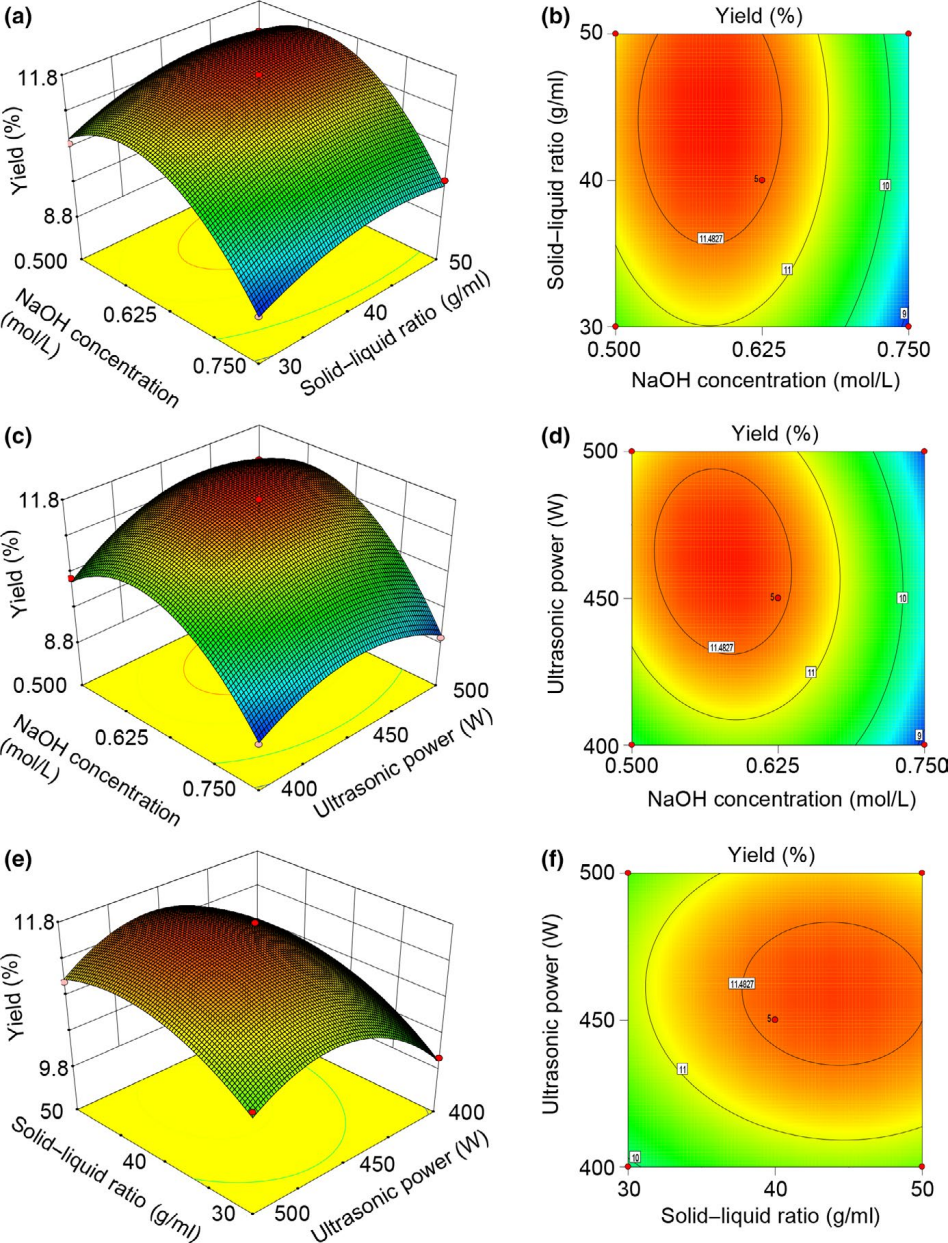
The 3D response surface and 2D contour plots showed the effects of extraction factors on the yield of melanin. (a, b) Solid–liquid ratio and NaOH concentration; (c, d) ultrasonic power and NaOH concentration; and (e, f) ultrasonic power and solid–liquid ratio

The response surface curves were described by the regression model constructed, and corresponding three‐dimensional response surfaces curves are shown in Figure 2. Each response surface plot illustrates the influence of two independent variables at an optimal level of the third variable. The optimal extraction conditions obtained by quadratic polynomial regression model were as follows: NaOH concentration was 0.58 mol/L, solid–liquid ratio was 1:44.01, and ultrasonic power was 461.82 W. Under optimum conditions, the predicted yield of melanin was 11.80%. Combined with the feasibility of practical operation, NaOH concentration of 0.58 mol/L, solid–liquid ratio of 1:44, and ultrasound power of 450 W were selected for verification experiments, and the yield of melanin was 11.99 ± 0.13%, which was in good agreement with the predicted value.

Ultrasound could produce strong vibration, cavitation effect, and agitation effect, thus promoting the dissolution of active components. In comparison with traditional extraction methods, ultrasonic‐assisted extraction can shorten the extraction time, improve the extraction efficiency, and reduce environmental pollution (Zhi, Xiaoxiang, & Jianrong, 2011). In this section, the ultrasound‐assisted extraction conditions of melanin from *A. auricula* waste residues were optimized by single‐factor experiment and RSM model, and the optimum process parameters were obtained to maximize the extraction rate of melanin. The results provide a reference for the comprehensive utilization of *A. auricula*.

### 3.3 Physicochemical properties of melanin from *Auricularia auricula* waste residue

**Figure 3:**
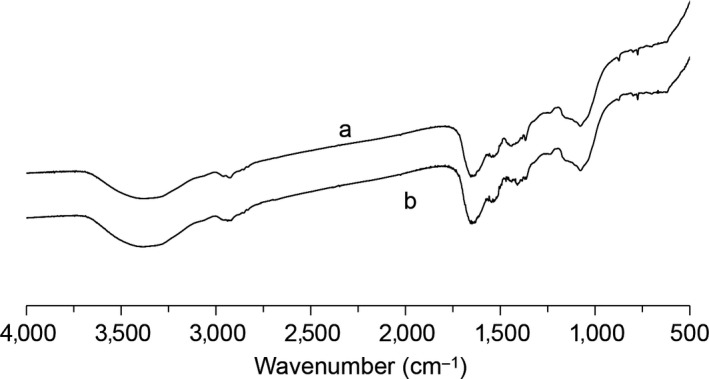
The IR spectrogram of melanin from (a) *Auricularia auricula* waste residue and (b) normal *A. auricula*

Melanin has typical characteristic absorption peaks in infrared spectra. The absorption peaks at about 3,300/cm correspond to the N‐H and O‐H groups in the melanin structure. The absorption peak at about 1,630/cm corresponds to C = O group or C = C group. The weak absorption band at 600–800/cm means that aromatic rings have been substituted to form conjugated systems. As shown in Figure 3, the main absorption peak of melanin from *A. auricula* waste residue and normal *A. auricula* are almost the same, which indicates that the structure of melanin extracted from the waste residue was almost unchanged. At the same time, the melanin obtained from the fruiting body of *A. auricula* (Figure 3) has a similar infrared absorption to the melanin obtained by liquid fermentation technique reported by sun et al (Sun, Zhang, Sun, et al., 2016). The results showed that the melanin produced by *A. auricula* in different growth states was similar in structure.

**Figure 4:**
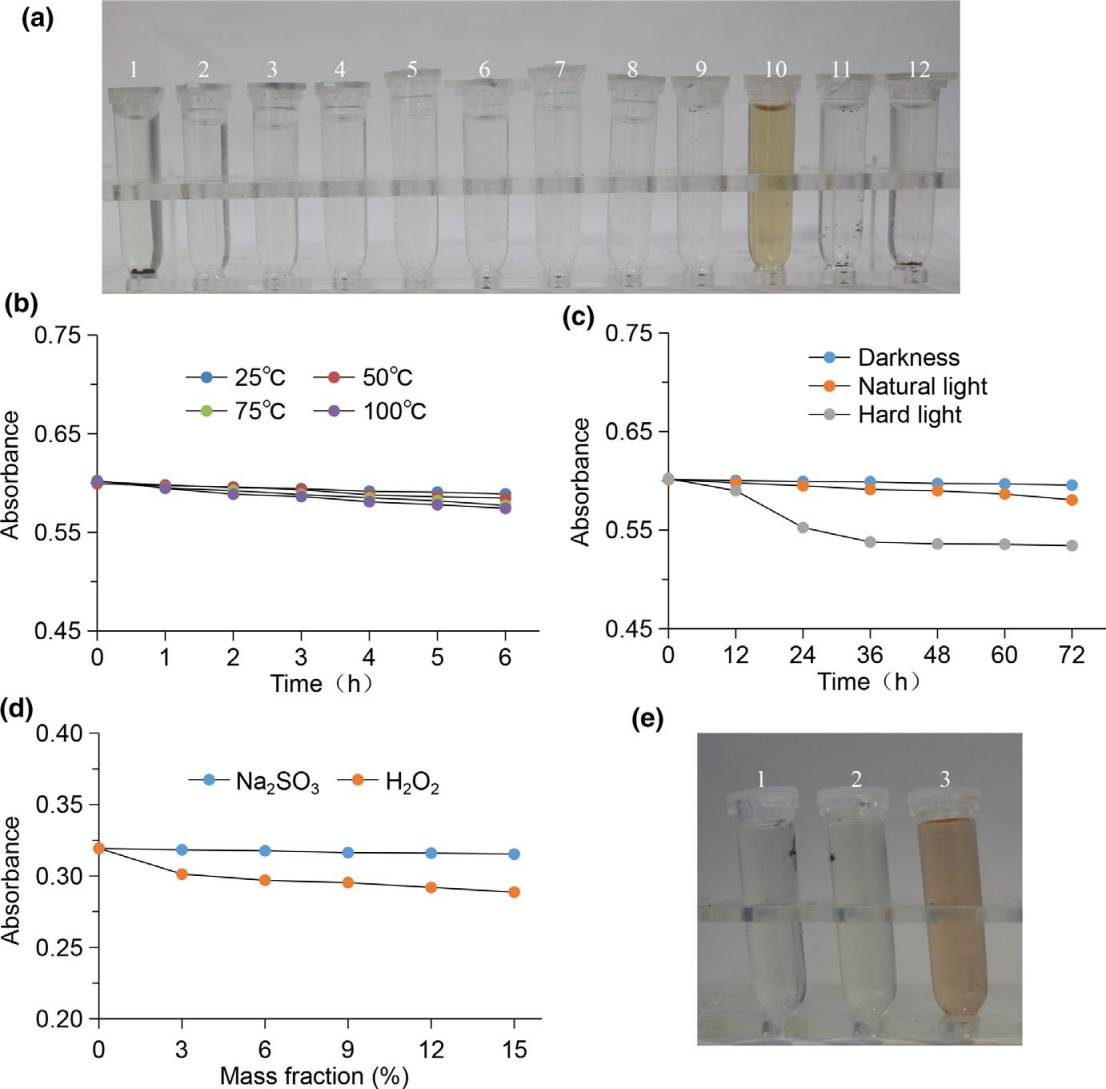
Physicochemical properties analysis of melanin from *Auricularia auricula* waste residue. (a) Solubility properties of melanin: The sequence number of each solvent in the test tube were distilled water, absolute ethanol, methanol, butanol, chloroform, dimethyl sulfoxide, petroleum ether, ethyl acetate, 1 mol/L HCl, 1 mol/L NaOH, 1 mol/L NaCl, and pH 8 disodium hydrogen phosphate–citrate buffer; (b) thermal stability of melanin; (c) photostability of melanin; (d) redox properties of melanin in Na_2_SO_3_ and H_2_O_2_; and (e) melanin bleached KMnO_4_ solution: The sequence number of each solvent in the test tube was 0.05 mg/ml melanin solution, 1 mg/ml KMnO_4_ + 0.05 mg/ml melanin solution, and 1 mg/ml KMnO_4_ solution

As shown in Table 4 and Figure 4a, the melanin has good solubility in alkaline solution (such as NaOH solution), but low solubility in distilled water and other organic solvents. The solubility of waste residue melanin was similar to the melanin that from other microorganisms (Kimura, Fukuda, Sanada, Fukuda, Sanada, & Imanaka, 2015; Madhusudhan et al., 2014). As shown in Figure 4b, the temperature had little effect on the stability of waste residue melanin. With the prolongation of time, the absorbance of melanin changed little in different temperature. As shown in Figure 4c, the stability of melanin to light was poor. The absorbance of melanin was basically unchanged with the prolongation of time under natural light, but decreased with the prolongation of time under strong light. The above results indicate that the melanin extracted from the fruit body waste residue of *A. auricula* had good thermal stability and a certain light resistance, which is consistent with the melanin in the fermentation broth of *A. auricula* reported by Wu et al (Wu, Zhang, Yang, Zhang, Yang, Zhou, & Yang, 2018). As shown in Figure 4d and e, the melanin had a certain stability in reductants such as Na_2_SO_3_, but the stability was poor in oxidants such as H_2_O_2_. The melanin also reacted with oxidants such as KMnO_4_, causing the discoloration of the solution. Instability in oxidants and relative stability in reductants are common characteristics of many melanins (Kumar, Mongolla, Pombala, Mongolla, Pombala, Kamle, & Joseph, 2011; Tan et al., 2011; Tu, Sun, Tian, Sun, Tian, Xie, & Chen, 2009; Yoon et al., 2003).

**Table 4:** Solubility of melanin from *Auricularia auricula* waste residue

Types	Solvent	Solubility
Water	Distilled water	−
Organic solvents	Absolute ethanol	−
	Methanol	−
	Butanol	−
	Chloroform	−
	Dimethyl sulfoxide	+
	Petroleum ether	−
	Ethyl acetate	−
Acid	1 mol/L HCl	−
Alkali	1 mol/L NaOH	+ +
Salt	1 mol/L NaCl	−
	pH8 Disodium hydrogen phosphate‐citrate buffer	+

Note

+ indicates slightly soluble, + + indicates dissolved, − indicates insoluble.

### 3.4 Antioxidant activity of melanin from *Auricularia auricula* waste residue

**Figure 5:**
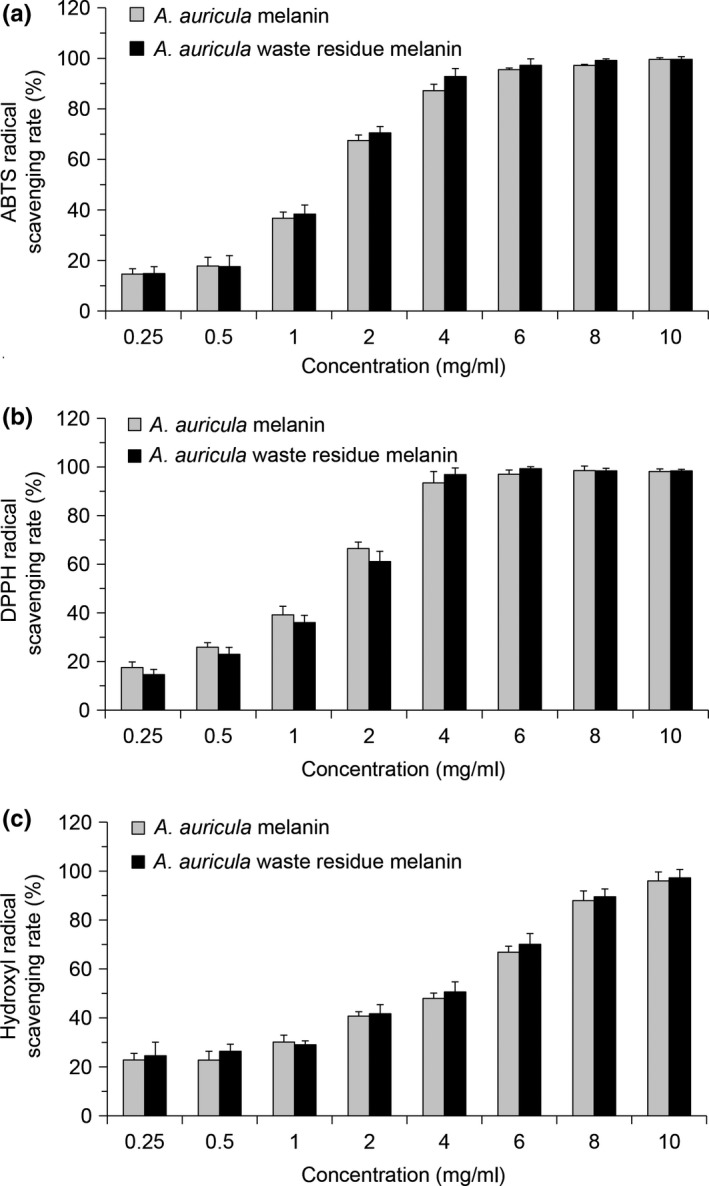
Antioxidant activity of melanin from *Auricularia auricula* waste residue and normal *A. auricula*. (a) ABTS radical scavenging activity; (b) DPPH radical scavenging activity; and (c) hydroxyl radical scavenging activity

The oxidation of ABTS produces relatively stable blue–green ABTS water‐soluble free radicals. The reaction of antioxidants with ABTS radicals will cause the solution fade and the decrease of characteristic absorbance, the more obvious of the solution discoloration, the stronger of the total antioxidant ability of the tested substance (Zhao et al., 2008). DPPH is a stable‐free radical in organic solvents. The alcohol solution of DPPH with a single electron appears purple, so it could accept one electron or hydrogen ion and have maximum absorption at a wavelength of 517 nm. In the presence of free radical scavenger, the single electron of DPPH is captured, which caused the discoloration of the solution and the decrease of the absorbance. DPPH has been widely used to determine the antioxidant capacity of biological samples and foods (Fogarasi, Kun, Tankó, Kun, Tankó, Stefanovits‐Bányai, & Hegyesné‐Vecseri, 2015; Kim, Lee, Lee, Lee, Lee, & Lee, 2002). Hydroxyl radical is one of the important active free radicals causing tissue cell damage. The human body produces hydroxyl radicals through metabolism in normal life activities. Hydroxyl radicals are thought to be the most toxic free radicals, which can cause membrane peroxidation, protein cross‐linking denaturation, and nucleic acid damage (Cacciuttolo, Trinh, Lumpkin, Trinh, Lumpkin, & Rao, 1993). As shown in Figure 5, *A. auricula* waste residue melanin has good antioxidant capacity, it has good scavenging activities to ABTS, DPPH, and hydroxyl radical, and its free radical scavenging activities were not significantly different from those of normal *A. auricula* melanin control group.

### 3.5 Protective effect of waste residue melanin on H_2_O_2_‐induced cell oxidative damage

**Figure 6:**
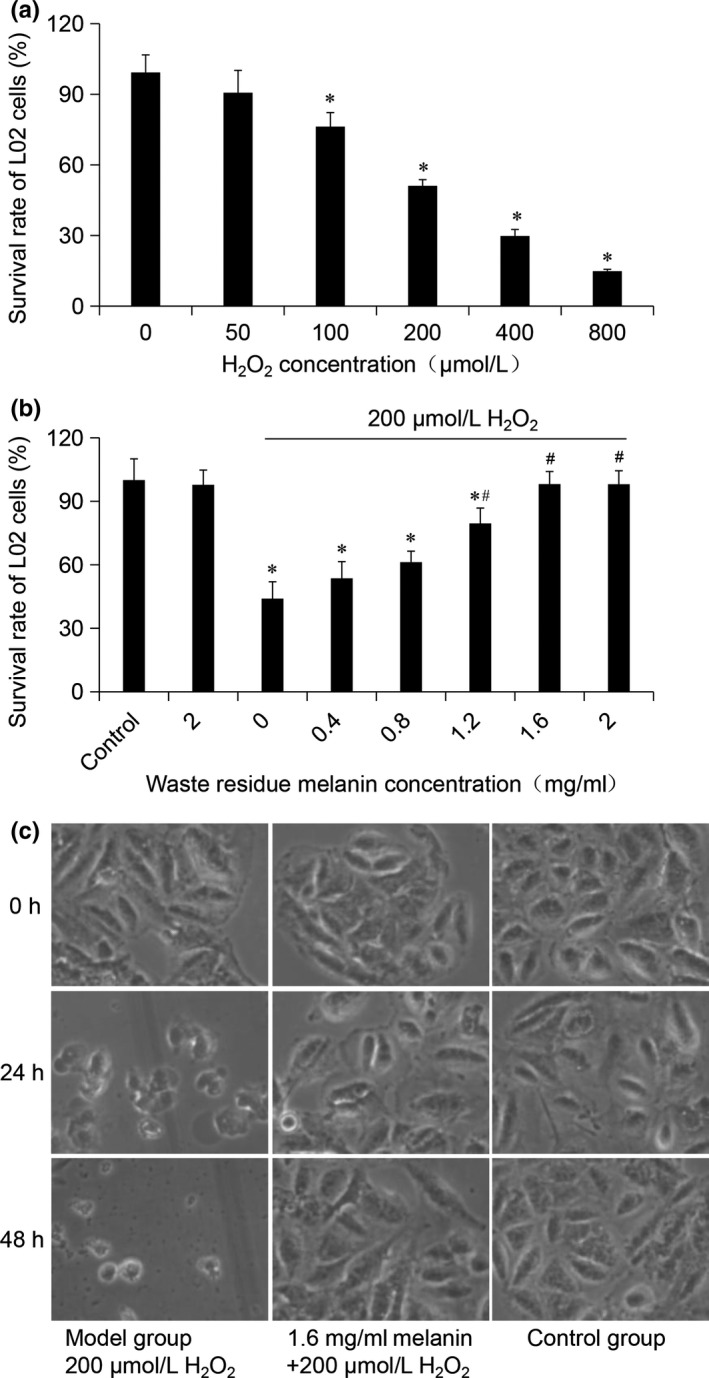
Protective effect of *Auricularia auricula* waste residue melanin on H_2_O_2_‐induced cell oxidative damage. (a) L02 cells were treated with different concentrations of H_2_O_2_ for 24 hr; (b) L02 cells were pretreated with *A. auricula* waste residue melanin for 1 hr followed by co‐incubated with 200 µM/L of H_2_O_2_ for 24 hr; and (c) observation of morphological changes of L02 cells by an inverted microscope. Data are presented as mean ± *SD*, *p < .05 and **p < .01 compared with control group; ^#^ *p* < .05 and ^##^ *p* < .01 compared with model group

The protective effect of melanin on H_2_O_2_‐induced cell oxidative damage was studied by MTT assay and morphology of the cells. First, different concentrations of H_2_O_2_ were given to human normal liver L02 cells. As shown in Figure 6a, when H_2_O_2_ concentration was 200 µM/L, the cell viability rate was 51.08 ± 2.65%. Therefore, 200 µM/L H_2_O_2_ was selected to induce oxidative damage of the cells in the further experiments. As shown in Figure 6b, the melanin dose‐dependently inhibited the decline of cell viability caused by H_2_O_2_, and the cell viability was 98.09 ± 5.97% when the concentration of melanin was 1.6 mg/ml, which indicated that the melanin significantly protected the cells from oxidative injury induced by H_2_O_2_. The effect of the melanin on the H_2_O_2_‐induced oxidative damage was further investigated by cell morphology observation. As shown in Figure 6c, compared with the blank control group, the cells in the model group gradually changed from fusiform to globular, the number of adherent cells decreased and showed apoptosis state with the prolongation of time. Compared with the model group, the melanin significantly ameliorated the effect of H_2_O_2_ on cell phenotype. Many studies have reported the good antioxidant effect of *A. auricula* polysaccharides (Xu, Zhang, & Jiang, 2016; Zhan, Xueping, Wenyan, Xueping, Wenyan, Xiaojuan, & Qi, 2015). In this study, we can conclude that in addition to *A. auricula* polysaccharides, its melanin component is also an excellent antioxidant.

## 4 CONCLUSIONS

*Auricularia auricula* is a popular edible and medicinal fungus in Eastern countries. Polysaccharides and melanin are two active components in *A. auricula*. However, most of the studies and related products about *A. auricula* are focused on its polysaccharides. After the extraction of *A. auricula* polysaccharides, the waste residue still contains melanin. Natural melanin can not only be used as a colorant, but also has many biological activities. Extracting melanin from the waste residue of *A. auricula* can make maximum use of *A. auricula* and reduce the waste of resources. In this study, the ultrasonic‐assisted extraction process of melanin from *A. auricula* waste residue was optimized, and the physicochemical properties and antioxidant activity of melanin were also studied. The results indicated that the optimal extraction parameters of melanin from the waste residue of *A. auricula* were the NaOH concentration of 0.58 mol/L, solid–liquid ratio of 1:44, ultrasonic power of 450 W, extraction time of 50 min, and extraction temperature of 70°C. Under the optimum conditions, the yield of melanin was 11.99 ± 0.13%, indicating that the ultrasound‐assisted extraction of *A. auricula* residue melanin is feasible. The melanin had a good thermal stability, but poor stability in strong light and oxidant. The IR spectra showed that the structure of waste residue melanin was basically the same as the normal *A. auricula* melanin. The study of antioxidant activity further proved that there was no significant difference between melanin from waste residue and normal *A. auricula* on ABTS, DPPH, and hydroxyl radical scavenging activity. Waste residue melanin also obviously ameliorated the H_2_O_2_‐induced oxidative damage of cells. This work provided a scientific basis for the comprehensive utilization of *A. auricula* waste residue, and the melanin could be used as excellent colorant and antioxidant in food products.

## CONFLICT OF INTEREST

We declare that we have no conflict of interest.

## ETHICAL APPROVAL

The uses of either humans or animals were not applicable in this study.
